# Supplementary material for: Concentration fluctuations in growing and dividing cells: Insights into the emergence of concentration homeostasis
Source: PLoS Comput Biol. 2022 Oct 4;18(10):e1010574. doi: 10.1371/journal.pcbi.1010574 (PMC9565450; doi:10.1371/journal.pcbi.1010574)
Supplement: S1 Appendix — This file contains the detailed derivation of some equations, the technical details of some figures, and the detailed description of the parameter inference method. (PDF) [file pcbi.1010574.s001.pdf]

S1 Appendix — Concentration fluctuations in growing and dividing cells: insights into the emergence of concentration homeostasis

Chen Jia, Abhyudai Singh, Ramon Grima

Contents

A Mean-field model of the concentration dynamics **1**

B Perfect concentration homeostasis **3**

C Derivation of the power spectrum expressions for the mean-field model **5**
C.1 Power spectrum of gene product number 5
C.2 Power spectrum of gene product concentration 6
C.3 Height of the off-zero peak for unstable gene products 10

D Technical details for Fig 4 **12**

E Technical details for Fig 5 **12**

F Technical details for Fig 6 **12**

G Parameter inference using synthetic data **13**

A. Mean-field model of the concentration dynamics

Note that the dynamics of gene product numbers at each cell cycle stage is the classical discrete bursty model proposed in [1]. It is interesting to understand the dynamics of gene product concentrations at each stage. To the end, we make the approximation of large molecule number. Recall that the burst size distribution $\xi = (\xi_n)$ is given by $\xi_n = p_B^n(1-p_B)$, where $p_B = B'/(B'+1)$ with $B' = BV(t)^\beta$ being the mean burst size. When the mean burst size B' is large, we have

$$-\log p_B = -\log(1 - 1/(B'+1)) \approx 1/(B'+1) = 1 - p_B$$

and thus the burst size distribution is given by

$$\xi_n = p_B^n(1-p_B) = e^{n\log p_B}(1-p_B) \approx e^{-(1-p_B)n}(1-p_B),$$

which is approximately an exponential distribution with mean $1/(1-p_B) = (B'+1) \approx B'$. As a result, the synthesis of the gene product at stage k can be described by a compound Poisson process with exponentially distributed interarrival times with rate ρ_k and exponentially distributed burst sizes with mean B'. By the scaling property of the exponential distribution, an exponentially distributed random variable with mean $B' = BV(t)^\beta$ can be viewed as an exponentially distributed random variable with mean B multiplied by $V(t)^\beta$. Therefore, in the

large burst size limit, the stochastic gene expression dynamics at stage k can be approximated by the stochastic differential equation (SDE) [2, 3]

$$\dot{n}(t) = V(t)^{\beta}\dot{s}_k(t) - dn(t), \tag{1}$$

where $n(t)$ denotes the gene product number at time t and $s_k(t)$ denotes a compound Poisson process with arrival rate ρ_k and an exponentially distributed jump distribution with mean B.

Let $c(t) = n(t)/V(t)$ denote the gene product concentration at time t. Then Eq. (1) can be written as

$$V(t)\dot{c}(t) + gV(t)c(t) = V(t)^{\beta}\dot{s}_k(t) - dV(t)c(t),$$

where we have used the fact that $\dot{V}(t) = gV(t)$ since cell volume grows exponentially with rate g. Thus the dynamics of gene product concentrations at stage k is governed by the SDE

$$\dot{c}(t) = V(t)^{\beta-1}\dot{s}_k(t) - (d+g)c(t) = V(t)^{\beta-1}\dot{s}_k(t) - d_{\text{eff}}c(t),$$

where $d_{\text{eff}} = d + g \approx d + \log(2)f$ is the effective decay rate of the gene product due to active degradation and dilution at cell division. Under the mean-field approximation, we have $V(t) \approx v_k$ and thus the concentration dynamics at stage k can be described by the continuous bursty model

$$\dot{c}(t) = v_k^{\beta-1}\dot{s}_k(t) - d_{\text{eff}}c(t). \tag{2}$$

The remaining question is whether cell division affects the concentration dynamics. Actually, in the large molecule number limit, the binomial partitioning of molecule number reduces to deterministic partitioning. This is a direct consequence of the law of large numbers since a binomial random variable can be viewed as the i.i.d. sum of Bernoulli random variables. At the moment of cell division, both the molecule number and the cell volume undergo deterministic symmetric partitioning, and thus their ratio remains invariant. This shows that in the large molecule number limit, cell division has no effect on concentration fluctuations.

Now we can construct a model describing concentration fluctuations across the cell cycle. At each cell cycle stage k, the concentration dynamics is governed by Eq. (2) and the system can hop from stage k to the next with rate q_k. The stochastic dynamics of this system can be described by a hybrid Markovian model whose Kolmogorov forward equation is given by [2, 4]

$$\begin{aligned}\partial_t p_1(x) &= d_{\text{eff}}\partial_x\big(xp(x)\big) + \rho_1\int_0^x w_1(x-y)p(y)dy - \rho_1 p(x)\\ &\quad + q_N p_N(x) - q_1 p_1(x),\\ \partial_t p_k(x) &= d_{\text{eff}}\partial_x\big(xp(x)\big) + \rho_k\int_0^x w_k(x-y)p(y)dy - \rho_k p(x)\\ &\quad + q_{k-1}p_{k-1}(x) - q_k p_k(x), \quad 2 \le k \le N,\end{aligned} \tag{3}$$

where $p_k(x)$ is the probability density of concentration when the cell is at stage k and

$$w_k(x) = \frac{1}{Bv_k^{\beta-1}}e^{-x/(Bv_k^{\beta-1})}$$

is the burst size distribution at stage k. A special case occurs when the synthesis is balanced ($\beta = 1$) and dosage compensation is perfect ($\kappa = 1$). In this case, both the burst frequency $\rho_k = \rho$ and the burst size distribution $w_k(x) = (1/B)e^{-x/B}$ are independent of cell cycle stage k, and thus the concentration dynamics along the whole cell lineage is governed by

$$\partial_t p(x) = d_{\text{eff}}\partial_x\big(xp(x)\big) + \rho\int_0^x w(x-y)p(y)dy - \rho p(x), \tag{4}$$

where $p(x)$ is the probability density of concentration. This is exactly the classical continuous bursty model proposed by Friedman et al. [5].

In summary, in the large burst size limit, the concentration dynamics of our model reduces to the classical continuous gene expression model when $\beta = \kappa = 1$. In this case, the steady-state distribution of concentration can be derived from Eq. (4) and is given by [5]

$$p(x) = \frac{1}{B^{\rho/d_{\text{eff}}}\Gamma(\rho/d_{\text{eff}})} x^{\rho/d_{\text{eff}}-1} e^{-x/B},$$

which is a gamma distribution. In this case, the concentration mean $\rho B/d_{\text{eff}}$ and variance $\rho B^2/d_{\text{eff}}$ are both independent of cell cycle stage and thus are both independent of cell volume.

B. Perfect concentration homeostasis

Concentration homeostasis is perfect when the mean concentration at each cell cycle stage is a constant. We have proved in Note 1 that when $\beta = \kappa = 1$, the mean and variance of concentration fluctuations are the same across the cell cycle provided the number of gene product molecules is large. Here we will prove that perfect homeostasis is achieved when $\beta = \kappa = 1$, even when the number of gene product molecules is small.

Before studying perfect homeostasis, we note that the cell cycle duration T for the mean-field model is the independent sum of N exponentially distributed random variables with rates $q_1, \cdots, q_N$, respectively. Practically, this distribution is well approximated by an Erlang distribution [6]. The mean and variance of the cell cycle duration can be easily computed as

$$\langle T \rangle = \frac{1}{q_1} + \cdots + \frac{1}{q_N}, \quad \sigma_T^2 = \frac{1}{q_1^2} + \cdots + \frac{1}{q_N^2}.$$

If we use an Erlang distribution with shape parameter $\bar{N}$ and rate $\bar{a}$ to approximate this distribution, then $\bar{N}$ and $\bar{a}$ should satisfy

$$\frac{\bar{N}}{\bar{a}} = \langle T \rangle, \quad \frac{\bar{N}}{\bar{a}^2} = \sigma_T^2.$$

Thus the two parameters can be determined as

$$\bar{N} = \frac{\langle T \rangle^2}{\sigma_T^2}, \quad \bar{a} = \frac{\langle T \rangle}{\sigma_T^2} = \bar{N} f.$$

Note that for the timer strategy, i.e. $\alpha_0, \alpha_1 \to 0$, the transition rate between stages is a constant and thus the cell cycle duration is exactly Erlang distributed. The above discussion suggests that the mean-field model for an arbitrary size control strategy can be well approximated by a mean-field model for the timer strategy with $\bar{N}$ cell cycle stages and transition rate $\bar{a} = \bar{N} f$ between stages. The parameter N_0 for the effective timer model can then be determined as $\bar{N}_0 = w\bar{N}$, where $w = \log_2(v_{N_0+1}/v_1)$ is the proportion of cell cycle before replication. Therefore, in order to investigate an arbitrary size control strategy, we only need to investigate the timer strategy first and then replace the parameters N and N_0 in the effective timer model by $\bar{N}$ and $\bar{N}_0$, respectively.

We next examine perfect concentration homeostasis for the timer strategy. In this case, the transition rate between stages is a constant and we denote it by $\bar{a} = Nf$. Since both W_{00} and W_{11} are circular matrices, their eigenvalues can be computed explicitly. The eigenvalues of W_{00} are given by

$$\lambda_k = -\bar{a} + \bar{a}\omega_k, \quad 1 \le k \le N, \tag{5}$$

and the eigenvalues of W_{11} are given by

$$\lambda_{N+k} = -d - \bar{a} + 2^{-1/N}\bar{a}\omega_k, \quad 1 \le k \le N, \tag{6}$$

where $\omega_k = e^{2(k-1)\pi i/N}$ are all the Nth roots of unity. Since W_{00} is a normal matrix, it is easy to check that there exists a complex orthogonal matrix

$$R = \frac{1}{\sqrt{N}} \begin{pmatrix} 1 & 1 & \cdots & 1 \\ \omega_1 & \omega_2 & \cdots & \omega_N \\ \cdots & \cdots & \cdots & \cdots \\ \omega_1^{N-1} & \omega_2^{N-1} & \cdots & \omega_N^{N-1} \end{pmatrix},$$

such that W_{00} is diagonalized, i.e.

$$W_{00} = RD_{00}\bar{R}', \tag{7}$$

where $D_{00} = \mathrm{diag}(\lambda_1, \cdots, \lambda_N)$ and $\bar{R}'$ denotes the conjugate transpose of R. Similarly, W_{11} can be diagonalized as

$$W_{11} = MRD_{11}\bar{R}'M^{-1}, \tag{8}$$

where $D_{11} = \mathrm{diag}(\lambda_{N+1}, \cdots, \lambda_{2N})$ and M is the diagonal matrix given by

$$M = \mathrm{diag}(1, 2^{-1/N}, \cdots, 2^{-(N-1)/N}).$$

It is clear that the matrices V and M are related by

$$V = v_1 M^{-1}. \tag{9}$$

Let μ_{lk} be the unnormalized lth moment of gene product concentrations at stage k and let $\mu_l = (\mu_{lk})$ be the row vector whose components are the lth moments of concentrations at all stages. It is easy to see that

$$\mu_1 = m_1 V^{-1}, \quad \mu_2 = (m_1 + m_2)V^{-2},$$

where $V = \mathrm{diag}(v_1, \cdots, v_N)$ is the diagonal matrix whose diagonal entries are the typical cell volumes at all stages. For the timer strategy, the vector m_0 can be computed explicitly as

$$m_0 = \frac{1}{N}\mathbb{1}'. \tag{10}$$

Combining Eqs. (8), (9), and (10), the vector μ_1 can be rewritten as

$$\mu_1 = -m_0 W_{01} W_{11}^{-1} V^{-1} = -\frac{1}{Nv_1}\mathbb{1}' W_{01} MRD_{11}^{-1}\bar{R}'.$$

This can be written in components as

$$\mu_{1k} = -\frac{1}{Nv_1}\sum_{j,l=1}^{N}\frac{[W_{01}M]_{jj}R_{jl}\bar{R}_{kl}}{\lambda_{N+l}} = \frac{1}{Nv_1}\sum_{j,l=1}^{N}\frac{[W_{01}M]_{jj}R_{jl}\bar{R}_{kl}}{d + \bar{a} - 2^{-1/N}\bar{a}\omega_l},$$

where A_{jl} denotes the (j, l)-th entry of the matrix A. It is easy to check that

$$W_{01} = ST = \rho B v_1^{\beta}\mathrm{diag}(1, \cdots, 2^{\beta(N_0-1)/N}, \kappa 2^{\beta N_0/N}, \cdots, \kappa 2^{\beta(N-1)/N}).$$

When $\beta = \kappa = 1$, we have $W_{01} = \rho B v_1 M^{-1}$ and thus $W_{01}M = \rho B v_1 I$ is a constant multiple of the identity matrix. This clearly shows that

$$\mu_{1k} = \frac{\rho B}{N}\sum_{j,l=1}^{N}\frac{R_{jl}\bar{R}_{kl}}{d + \bar{a} - 2^{-1/N}\bar{a}\omega_l}. \tag{11}$$

We next make a crucial observation that

$$\sum_{j=1}^{N} R_{jl} = \frac{1}{\sqrt{N}}\sum_{j=1}^{N}\omega_l^{j-1} = \sqrt{N}\delta_{l1}.$$

Inserting this equation into Eq. (11) yields

$$\mu_{1k} = \frac{\rho B}{\sqrt{N}}\frac{\bar{R}_{k1}}{d + \bar{a} - 2^{-1/N}\bar{a}\omega_1} = \frac{\rho B}{N(d + \bar{a} - 2^{-1/N}\bar{a})}.$$

Note that when $N \gg 1$, we have

$$d + \bar{a} - 2^{-1/N}\bar{a} = d + (1 - e^{-\log(2)/N})Nf \approx d + (\log 2)f = d_{\mathrm{eff}}.$$

Therefore, the unnormalized mean concentration μ_{1k} at stage k is given by

$$\mu_{1k} = \frac{\rho B}{N d_{\text{eff}}},$$

and the normalized mean concentration μ_{1k} at stage k is given by

$$\mu_k = \frac{\mu_{1k}}{m_{0k}} = \frac{\rho B}{d_{\text{eff}}},$$

which is independent of stage k. As a result, we have proved that perfect concentration homeostasis is achieved when $\beta = \kappa = 1$.

C. Derivation of the power spectrum expressions for the mean-field model

C.1 Power spectrum of gene product number

Let $r(t)$ denote the cell cycle stage and let $n(t)$ denote the gene product number in a single cell at time t. To proceed, let

$$\begin{aligned}
m_{0k}(t) &= \sum_{n=0}^{\infty} p_{k,n} = \mathbb{P}(r(t) = k), \\
m_{1k}(t) &= \sum_{n=0}^{\infty} n p_{k,n} = \mathbb{E} n(t) I_{\{r(t)=k\}}, \\
m_{2k}(t) &= \sum_{n=0}^{\infty} n(n-1) p_{k,n} = \mathbb{E} n(t)(n(t)-1) I_{\{r(t)=k\}},
\end{aligned}$$

be the zeroth, first, and second factorial moments of gene product numbers at stage k, where I_A denotes the indicator function of the set A. For convenience, let $m_k(t) = (m_{kr}(t))$ be the row vector whose components are the kth factorial moments at all stages. It then follows from Eq. (6) in the main text that $m_0(t)$, $m_1(t)$, and $m_2(t)$ satisfy the following differential equations:

$$\begin{aligned}
\dot{m}_0(t) &= m_0(t) W_{00}, \\
\dot{m}_1(t) &= m_1(t) W_{11} + m_0(t) W_{01}, \\
\dot{m}_2(t) &= m_2(t) W_{22} + m_1(t) W_{12} + m_0(t) W_{02}.
\end{aligned} \tag{12}$$

Since Eq. (12) is a set of linear differential equations, its time-dependent solution is given by

$$m_1(t) = m_1(0) e^{W_{11} t} + \int_0^t m_0(0) e^{W_{00} s} W_{01} e^{W_{11}(t-s)} \mathrm{d}s. \tag{13}$$

Given the initial cell cycle stage $r(0) = k$ and initial gene product number $n(0) = n$, it follows that

$$\mathbb{E} n(t) = m_1(t) \mathbb{1} = n e_k e^{W_{11} t} \mathbb{1} + \int_0^t e_k e^{W_{00} s} W_{01} e^{W_{11}(t-s)} \mathbb{1} \mathrm{d}s,$$

where e_k denotes the row vector whose kth component is 1 and all other components are 0. This clearly shows that

$$\mathbb{E}[n(t) | r(0), n(0)] = n(0) e_{r(0)} e^{W_{11} t} \mathbb{1} + \int_0^t e_{r(0)} e^{W_{00} s} W_{01} e^{W_{11}(t-s)} \mathbb{1} \mathrm{d}s.$$

From now on, we assume that the system has reached the steady state. Then we have

$$
\begin{aligned}
&\mathbb{E}n(0)n(t) \\
&= \sum_k \mathbb{E}n(0)I_{\{r(0)=k\}}\mathbb{E}[n(t)|r(0),n(0)] \\
&= \sum_k \mathbb{E}n(0)I_{\{r(0)=k\}}\left[n(0)e_{r(0)}e^{W_{11}t}\mathbb{1} + \int_0^t e_{r(0)}e^{W_{00}s}W_{01}e^{W_{11}(t-s)}\mathbb{1}\mathrm{d}s\right] \\
&= \sum_k \mathbb{E}n(0)^2I_{\{r(0)=k\}}e_ke^{W_{11}t}\mathbb{1} + \int_0^t \mathbb{E}n(0)I_{\{r(0)=k\}}e_ke^{W_{00}s}W_{01}e^{W_{11}(t-s)}\mathbb{1}\mathrm{d}s \\
&= \sum_k (m_{1k}+m_{2k})e_ke^{W_{11}t}\mathbb{1} + \int_0^t m_{1k}e_ke^{W_{00}s}W_{01}e^{W_{11}(t-s)}\mathbb{1}\mathrm{d}s \\
&= (m_1+m_2)e^{W_{11}t}\mathbb{1} + \int_0^t m_1e^{W_{00}s}W_{01}e^{W_{11}(t-s)}\mathbb{1}\mathrm{d}s,
\end{aligned}
$$

where $m_1 = -m_0STW_{11}^{-1}$ and $m_2 = -2(m_1ST + m_0ST^2)W_{22}^{-1}$ are the steady-state values of the first and second moments. Since the autocorrelation function is defined as $R_n(t) = \mathbb{E}n(0)n(t) - \mathbb{E}n(0)\mathbb{E}n(t)$, we finally obtain an explicit expression of the autocorrelation function, which is given by

$$
R_n(t) = (m_1+m_2)e^{W_{11}t}\mathbb{1} + \int_0^t m_1e^{W_{00}s}W_{01}e^{W_{11}(t-s)}\mathbb{1}\mathrm{d}s - (m_1\mathbb{1})^2.
$$

C.2 Power spectrum of gene product concentration

Let $c(t)$ denote the gene product concentration in a single cell at time t. To proceed, let

$$
\begin{aligned}
\mu_{1k}(t) &= \mathbb{E}c(t)I_{\{r(t)=k\}} = m_{1k}(t)/v_k, \\
\mu_{2k}(t) &= \mathbb{E}c(t)^2I_{\{r(t)=k\}} = (m_{1k}(t)+m_{2k}(t))/v_k^2,
\end{aligned}
$$

be the first and second moments of concentration at stage k. For convenience, let $\mu_k(t) = (\mu_{kr}(t))$ denote the row vector whose components are the kth moments of concentration at all stages. It is easy to see that

$$
\mu_1(t) = m_1(t)V^{-1}, \quad \mu_2(t) \approx [m_1(t)+m_2(t)]V^{-2}.
$$

It then follows from Eq. (12) that

$$
\mu_1(t) = m_1(0)e^{W_{11}t}V^{-1} + \int_0^t m_0(0)e^{W_{00}s}W_{01}e^{W_{11}(t-s)}V^{-1}\mathrm{d}s.
$$

Given the initial cell cycle stage $r(0) = k$ and initial gene product number $n(0) = n$, it follows that

$$
\mathbb{E}c(t) = \mu_1(t)\mathbb{1} = ne_ke^{W_{11}t}V^{-1}\mathbb{1} + \int_0^t e_ke^{W_{00}s}W_{01}e^{W_{11}(t-s)}V^{-1}\mathbb{1}\mathrm{d}s.
$$

This clearly shows that

$$
\mathbb{E}[c(t)|r(0),n(0)] \approx n(0)e_{r(0)}e^{W_{11}t}V^{-1}\mathbb{1} + \int_0^t e_{r(0)}e^{W_{00}s}W_{01}e^{W_{11}(t-s)}V^{-1}\mathbb{1}\mathrm{d}s.
$$

From now on, we assume that the system has reached the steady state. Then we have

$$\begin{aligned}
&\mathbb{E}c(0)c(t)\\
&=\sum_k \mathbb{E}c(0)I_{\{r(0)=k\}}\mathbb{E}[c(t)|r(0),n(0)]\\
&=\sum_k \mathbb{E}c(0)I_{\{r(0)=k\}}\left[n(0)e_{r(0)}e^{W_{11}t}V^{-1}\mathbb{1}+\int_0^t e_{r(0)}e^{W_{00}s}W_{01}e^{W_{11}(t-s)}V^{-1}\mathbb{1}\mathrm{d}s\right]\\
&=\sum_k \mathbb{E}c(0)^2 I_{\{r(0)=k\}}v_k e_k e^{W_{11}t}V^{-1}\mathbb{1}+\int_0^t \mathbb{E}c(0)I_{\{r(0)=k\}}e_k e^{W_{00}s}W_{01}e^{W_{11}(t-s)}V^{-1}\mathbb{1}\mathrm{d}s\\
&=\sum_k \mu_{2k}v_k e_k e^{W_{11}t}V^{-1}\mathbb{1}+\int_0^t \mu_{1k}e_k e^{W_{00}s}W_{01}e^{W_{11}(t-s)}V^{-1}\mathbb{1}\mathrm{d}s\\
&=\mu_2 V e^{W_{11}t}V^{-1}\mathbb{1}+\int_0^t \mu_1 e^{W_{00}s}W_{01}e^{W_{11}(t-s)}V^{-1}\mathbb{1}\mathrm{d}s\\
&=(m_1+m_2)V^{-1}e^{W_{11}t}V^{-1}\mathbb{1}+\int_0^t m_1V^{-1}e^{W_{00}s}W_{01}e^{W_{11}(t-s)}V^{-1}\mathbb{1}\mathrm{d}s,
\end{aligned}$$

where $m_1=-m_0STW_{11}^{-1}$ and $m_2=-2(m_1ST+m_0ST^2)W_{22}^{-1}$ are the steady-state values of the first and second moments. Since the autocorrelation function is defined as $R_c(t)=\mathbb{E}c(0)c(t)-\mathbb{E}c(0)\mathbb{E}c(t)$, we finally obtain the explicit expression of the autocorrelation function, which is given by

$$R_c(t)=(m_1+m_2)V^{-1}e^{W_{11}t}V^{-1}\mathbb{1}+\int_0^t m_1V^{-1}e^{W_{00}s}W_{01}e^{W_{11}(t-s)}V^{-1}\mathbb{1}\mathrm{d}s-(m_1V^{-1}\mathbb{1})^2. \tag{14}$$

Here the autocorrelation function is expressed in matrix form. A more explicit expression can be obtained by expanding the matrix exponentials $e^{W_{11}t}$ and $e^{W_{00}s}$ in terms of their eigenvalues and eigenvectors. For simplicity, we next focus on the timer strategy. The results for other control strategies can be obtained from the results for the timer strategies by substituting the parameters N and N_0 for the effective parameters $\bar{N}$ and $\bar{N}_0$, respectively (see Sec. B for details). With these notation in Sec. B, the autocorrelation function given in Eq. (14) can be rewritten as

$$R_c(t)=\frac{1}{v_1^2}\left[(m_1+m_2)M^2Re^{D_{11}t}\bar{R}'\mathbb{1}+\int_0^t m_1MRe^{D_{00}s}\bar{R}'W_{01}MRe^{D_{11}(t-s)}\bar{R}'\mathbb{1}\mathrm{d}s-(m_1M\mathbb{1})^2\right].$$

This suggests that the autocorrelation function (power spectrum) is actually the linear combination of $2N-1$ exponential (Lorentzian) functions:

$$R_c(t)=\sum_{k=2}^{2N}u_ke^{\lambda_kt},\quad G_c(\xi)=\sum_{k=2}^{2N}\frac{-2u_k\lambda_k}{4\pi^2\xi^2+\lambda_k^2},$$

where $\lambda_1,\cdots,\lambda_{2N}$ are all the eigenvalues of W_{00} and W_{11}, all the coefficients u_k associated with the eigenvalues of W_{00} are given by

$$u_k=\frac{1}{v_1^2}\sum_{j=1}^N\frac{[m_1MR]_k[\bar{R}'W_{01}MR]_{kj}[\bar{R}'\mathbb{1}]_j}{\lambda_k-\lambda_{N+j}},\quad 2\leq k\leq N,$$

and all the coefficients u_k associated with the eigenvalues of W_{11} are given by

$$u_{N+k}=\frac{1}{v_1^2}[(m_1+m_2)M^2R]_k[\bar{R}'\mathbb{1}]_k-\frac{1}{v_1^2}\sum_{j=1}^N\frac{[m_1MR]_j[\bar{R}'W_{01}MR]_{jk}[\bar{R}'\mathbb{1}]_k}{\lambda_j-\lambda_{N+k}},\quad 1\leq k\leq N.$$

Here $[m]_k$ denotes the kth entry of the vector m and A_{kl} denotes the (k,j)-th entry of the matrix A. In fact, the coefficients u_k can be computed more explicitly. To see this, note that

$$[\bar{R}'\mathbb{1}]_k=\frac{1}{\sqrt{N}}\sum_{j=1}^N[\bar{R}]_{jk}=\frac{1}{\sqrt{N}}\sum_{j=1}^N\bar{\omega}_k^{j-1}=\sqrt{N}\delta_{k1}.$$

Thus all the coefficients u_k associated with the eigenvalues of W_{00} can be simplified as

$$u_k = \frac{\sqrt{N}}{v_1^2}\frac{[m_1MR]_k[\bar{R}'W_{01}MR]_{k1}}{\lambda_k-\lambda_{N+1}}, \quad 1\leq k\leq N, \tag{15}$$

and all the coefficients u_k associated with the eigenvalues of W_{11} can be simplified as

$$\begin{aligned} &u_{N+k} = 0, \quad 2\leq k\leq N,\\ &u_{N+1} = \frac{\sqrt{N}}{v_1^2}[(m_1+m_2)M^2R]_1 - \frac{\sqrt{N}}{v_1^2}\sum_{j=1}^N\frac{[m_1MR]_j[\bar{R}'W_{01}MR]_{j1}}{\lambda_j-\lambda_{N+1}}. \end{aligned} \tag{16}$$

This suggests that the autocorrelation function (power spectrum) is actually the linear combination of only N exponential (Lorentzian) functions:

$$R_c(t) = \sum_{k=2}^{N+1}u_ke^{\lambda_kt}, \quad G_c(\xi) = \sum_{k=2}^{N+1}\frac{-2u_k\lambda_k}{4\pi^2\xi^2+\lambda_k^2},$$

Since $m_1 = -m_0STW_{11}^{-1}$, it follows from Eqs. (8) and (10) that

$$m_1MR = -m_0W_{01}W_{11}^{-1}MR = -\frac{1}{N}\mathbb{1}'W_{01}MRD_{11}^{-1}.$$

It is easy to check that

$$W_{01} = ST = \rho Bv_1^\beta\mathrm{diag}(1,\cdots,2^{\beta(N_0-1)/N},\kappa2^{\beta N_0/N},\cdots,\kappa2^{\beta(N-1)/N}).$$

This shows that

$$\begin{aligned} [m_1MR]_k &= -\frac{1}{N}\sum_{j=1}^N[W_{01}M]_{jj}R_{jk}[D_{11}^{-1}]_{kk} = -\frac{1}{N^{3/2}\lambda_{N+k}}\sum_{j=1}^N[W_{01}M]_{jj}\omega_k^{j-1}\\ &= \frac{\rho Bv_1^\beta}{N^{3/2}(d+a-2^{-1/N}a\omega_k)}\left[\sum_{j=1}^{N_0}2^{(\beta-1)(j-1)/N}\omega_k^{j-1}+\kappa\sum_{j=N_0+1}^N2^{(\beta-1)(j-1)/N}\omega_k^{j-1}\right]\\ &= \frac{\rho Bv_1^\beta}{N^{3/2}(d+a-2^{-1/N}a\omega_k)}\left[\sum_{j=1}^{N_0}(2^{(\beta-1)/N}\omega_k)^{j-1}+\kappa\sum_{j=N_0+1}^N(2^{(\beta-1)/N}\omega_k)^{j-1}\right]\\ &= \frac{\rho Bv_1^\beta\Delta_k}{N^{3/2}(d+a-2^{-1/N}a\omega_k)}, \end{aligned}$$

where

$$\begin{aligned} \Delta_k &= \sum_{j=1}^{N_0}(2^{(\beta-1)/N}\omega_k)^{j-1}+\kappa\sum_{j=N_0+1}^N(2^{(\beta-1)/N}\omega_k)^{j-1}\\ &= \frac{1-\kappa2^{(\beta-1)}+(\kappa-1)2^{(\beta-1)w}\omega_k^{N_0}}{1-2^{(\beta-1)/N}\omega_k}, \end{aligned}$$

with $w = N_0/N$ being the proportion of cell cycle before replication. Moreover, we have

$$[\bar{R}'W_{01}MR]_{k1} = \sum_{j=1}^N[\bar{R}]_{jk}[W_{01}M]_{jj}[R]_{j1} = \frac{1}{N}\sum_{j=1}^N\bar{\omega}_k^{j-1}[W_{01}M]_{jj} = \frac{\rho Bv_1^\beta\bar{\Delta}_k}{N}.$$

It then follows from Eq. (15) that

$$u_k = \frac{\rho^2B^2v_1^{2\beta-2}|\Delta_k|^2}{N^2(d+a-2^{-1/N}a\omega_k)(d+a\omega_k-2^{-1/N}a)}, \quad 1\leq k\leq N.$$

In addition, it is easy to see that

$$[(m_1+m_2)M^2R]_1 = \sum_{j=1}^{N}[(m_1+m_2)M^2]_j R_{j1} = \frac{1}{\sqrt{N}}(m_1+m_2)M^2\mathbb{1}.$$

It thus follows from Eq. (16) that

$$u_{N+1} = \frac{1}{v_1^2}(m_1+m_2)M^2\mathbb{1} - \sum_{k=1}^{N} u_k = \langle c(t)\rangle^2 - \sum_{k=1}^{N} u_k.$$

To proceed, note that the concentration mean can be computed explicitly as

$$\begin{aligned}
\langle c(t)\rangle &= m_1V^{-1}\mathbb{1} = -\frac{1}{Nv_1}\mathbb{1}'W_{01}W_{11}^{-1}M\mathbb{1} = -\frac{1}{Nv_1}\mathbb{1}'W_{01}MRD_{11}^{-1}\bar{R}'\mathbb{1} \\
&= \frac{1}{Nv_1}\sum_{j,k=1}^{N}\frac{[W_{01}M]_{jj}R_{jk}[\bar{R}'\mathbb{1}]_k}{\lambda_{N+k}} \\
&= \frac{1}{Nv_1(d+a-2^{-1/N}a)}\sum_{j=1}^{N}[W_{01}M]_{jj} \\
&= \frac{\rho B v_1^{\beta-1}\Delta_1}{N(d+a-2^{-1/N}a)} = \sqrt{u_1}.
\end{aligned}$$

Thus we obtain

$$u_{N+1} = \langle c(t)\rangle^2 - \langle c(t)\rangle^2 - \sum_{k=2}^{N} u_k = \sigma_c^2 - \sum_{k=2}^{N} u_k,$$

where σ_c^2 is the steady-state variance of gene product concentration.

In summary, we have proved that the autocorrelation function (power spectrum) is the weighted sum of N exponential (Lorentzian) functions:

$$R_c(t) = \sum_{k=1}^{N} u_k e^{\lambda_k t}, \quad G_c(\xi) = \sum_{k=1}^{N}\frac{-2u_k\lambda_k}{4\pi^2\xi^2+\lambda_k^2}, \tag{17}$$

where the exponents λ_k are given by

$$\begin{aligned}
&\lambda_k = -a(1-\omega_k), \quad 1\le k\le N-1, \\
&\lambda_N = -d-a+2^{-1/N}a = -d-a(1-e^{-\log(2)/N}) \approx -d-\log(2)f = -d_{\text{eff}},
\end{aligned}$$

with $\omega_k = e^{2k\pi i/N}$ being all Nth roots of unity, and the coefficients u_k are given by

$$\begin{aligned}
u_k &= \frac{\rho^2B^2v_1^{2\beta-2}|\Delta_k|^2}{N^2(d+a-2^{-1/N}a\omega_k)(d+a\omega_k-2^{-1/N}a)}, \quad 1\le k\le N-1, \\
u_N &= \sigma_c^2 - \sum_{k=1}^{N-1} u_k,
\end{aligned}$$

with σ_c^2 being the steady-state variance of concentration and with Δ_k being defined as

$$\Delta_k = \frac{1-\kappa 2^{(\beta-1)}+(\kappa-1)2^{(\beta-1)w}\omega_k^{N_0}}{1-2^{(\beta-1)/N}\omega_k}. \tag{18}$$

In the special case of perfect homeostasis ($\beta=\kappa=1$), we have $\Delta_k = u_k = 0$ for each $1\le k\le N-1$ and thus the autocorrelation function (power spectrum) reduces to the following exponential (Lorentzian) function:

$$R_c(t) = \sigma_c^2 e^{-d_{\text{eff}}t}, \quad G_c(\xi) = \frac{-2\sigma_c^2 d_{\text{eff}}}{4\pi^2\xi^2+d_{\text{eff}}^2}.$$

In this case, both the autocorrelation function and power spectrum are monotonic decreasing functions and thus no concentration oscillations can be observed.

If homeostasis is not perfect, the power spectrum for concentration can either be monotonically decreasing or have an off-zero peak. When $N \gg 1$, the position of the off-zero peak is equal to the cell cycle frequency f, the width of the off-zero peak is given by $D = 2\pi f/N$. The absolute height of the zero peak is given by

$$H_{\mathrm{zero}} = G_c(0) = -2\sum_{k=1}^{N} \frac{u_k}{\lambda_k}.$$

Moreover, the absolute height of the off-zero peak is given by

$$H_{\mathrm{off-zero}} = \frac{-2u_1\lambda_1}{4\pi^2 f^2 + \lambda_1^2} + \frac{-2u_1\lambda_1}{4\pi^2 f^2 + \lambda_1^2} = -4\mathrm{Re}\left(\frac{u_1\lambda_1}{4\pi^2 f^2 + \lambda_1^2}\right),$$

where $\mathrm{Re}(z)$ denotes the real part of z. Since we have normalized the power spectrum so that $G_c(0) = 1$, the height of the off-zero peak is then the ratio of the absolute heights of the off-zero and zero peaks, which is given by

$$H = \frac{H_{\mathrm{zero}}}{H_{\mathrm{off-zero}}} = \frac{2\mathrm{Re}(u_1\lambda_1/(4\pi^2 f^2 + \lambda_1^2))}{\sum_{k=1}^{N} u_k/\lambda_k}. \tag{19}$$

Note that the height H is proportional to u_1, which is proportional to $|\Delta_1|^2$. It follows from Eq. (18) that $|\Delta_1|^2$ is proportional to

$$C_1(w,\kappa,\beta) = |1 - \kappa 2^{(\beta-1)} + (\kappa-1)2^{(\beta-1)w}\omega_1^{N_0}|^2 = |1 - \kappa 2^{(\beta-1)} + (\kappa-1)2^{(\beta-1)w}e^{2\pi wi}|^2.$$

Therefore, the height H is also proportional to $C_1(w,\kappa,\beta)$ and thus vanishes if $C_1(w,\kappa,\beta) = 0$.

C.3 Height of the off-zero peak for unstable gene products

Here we focus on the power spectrum for unstable gene products. Without loss of generality, we assume that at least one of β and κ is not equal to 1. For unstable gene products, we have $d \gg a$ and thus $|\lambda_{N+1}| \gg |\lambda_k|$ for $2 \le k \le N$. Thus the power spectrum of gene product concentrations is approximately given by

$$G_c(\xi) = \sum_{k=1}^{N-1} \frac{-2u_k\lambda_k}{4\pi^2\xi^2 + \lambda_k^2},$$

where $\lambda_k = -a + a\omega_k$ with $\omega_k = e^{2k\pi i/N}$ and

$$u_k = \frac{\rho^2 B^2 v_1^{2\beta-2}|\Delta_k|^2}{a^2\eta^2}.$$

Note that the power spectrum can be rewritten as

$$G_c(\xi) = \frac{2\rho^2 B^2 v_1^{2\beta-2}}{a\eta^2}\sum_{k=1}^{N-1} \frac{|\Delta_k|^2(1-\omega_k)}{4\pi^2\xi^2 + a^2(1-\omega_k)^2}.$$

When $N \gg 1$, the power spectrum has the following approximation:

$$G_c(\xi) \approx \frac{2\rho^2 B^2 v_1^{2\beta-2}}{a\eta^2}\sum_{k=1}^{[N/2]} |\Delta_k|^2 G_k(\xi),$$

where

$$G_k(\xi) = \frac{1-\omega_k}{4\pi^2\xi^2 + a^2(1-\omega_k)^2} + \frac{1-\bar{\omega}_k}{4\pi^2\xi^2 + a^2(1-\bar{\omega}_k)^2}.$$

Straightforward computations show that

$$G_k(\xi) = \frac{\sin^2\theta_k(\pi^2\xi^2 + a^2\sin^2\theta_k)}{(\pi^2\xi^2 - a^2\sin^2\theta_k)^2 + 4\pi^2\xi^2a^2\sin^4\theta_k},$$

where $\theta_k = k\pi/N$. In fact, the function $G_k(\xi)$ characterizes the kth off-zero peak. It is easy to check that the position of the kth off-zero peak is given by

$$\xi = \frac{a}{\pi}\sin\theta_k\sqrt{2\cos\theta_k - 1}.$$

When $N \gg 1$, we have $\sin\theta_k \approx \theta_k$ and $\cos\theta_k \approx 1$ and thus the position of the kth off-zero peak is approximately given by

$$\xi = \frac{a\theta_k}{\pi} \approx kf,$$

and the function $G_k(\xi)$ can be further simplified as

$$G_k(\xi) \approx \frac{k^2(\xi^2 + k^2f^2)}{N^2(\xi^2 - k^2f^2)^2 + 4k^4\pi^2f^2\xi^2}. \tag{20}$$

In addition, we have

$$|\Delta_k|^2 = \frac{|1 - \kappa 2^{(\beta-1)} + (\kappa-1)2^{(\beta-1)w}\omega_k^{N_0}|^2}{|1 - 2^{(\beta-1)/N}\omega_k|^2} = \frac{C_k(w,\kappa,\beta)}{|1 - 2^{(\beta-1)/N}\omega_k|^2},$$

where

$$C_k(w,\kappa,\beta) = 2^{2(\beta-1)w}(\kappa-1)^2 + (1-\kappa 2^{\beta-1})^2 + 2^{(\beta-1)w+1}(\kappa-1)(1-\kappa 2^{\beta-1})\cos(2k\pi w)$$

is a function of w, κ, and β. Moreover, note that

$$\begin{aligned}|1 - 2^{(\beta-1)/N}\omega_k|^2 &= (1 - 2^{(\beta-1)/N})^2 + 2^{(\beta-1)/N+2}\sin^2\theta_k \\ &\approx \frac{4k^2\pi^2 + (\log 2)^2(\beta-1)^2}{N^2}.\end{aligned}$$

Thus we have

$$|\Delta_k|^2 \approx \frac{C_k(w,\kappa,\beta)N^2}{4k^2\pi^2 + (\log 2)^2(\beta-1)^2}.$$

Thus when $N \gg 1$, the power spectrum for unstable gene products can be simplified as

$$G_c(\xi) \approx \frac{2\rho^2B^2v_1^{2\beta-2}N}{f\eta^2}\sum_{k=1}^{\infty}\frac{C_k(w,\kappa,\beta)}{4k^2\pi^2 + (\log 2)^2(\beta-1)^2}G_k(\xi),$$

where $G_k(\xi)$ is given in Eq. (20). Note that the maximum of $G_k(\xi)$ is approximately given by

$$G_k(kf) \approx \frac{2k^4f^2}{4k^6\pi^2f^4} = \frac{1}{2k^2\pi^2f^2}.$$

Thus the absolute height of the off-zero peak is given by

$$H_{\text{off-zero}} \approx \frac{2\rho^2B^2v_1^{2\beta-2}N}{f\eta^2} \times \frac{C_1(w,\kappa,\beta)}{4\pi^2 + (\log 2)^2(\beta-1)^2} \times \frac{1}{2\pi^2f^2}.$$

Moreover, the absolute height of the zero peak is given by

$$\begin{aligned}H_{\text{zero}} &\approx \frac{2\rho^2B^2v_1^{2\beta-2}N}{f\eta^2}\sum_{k=1}^{\infty}\frac{C_k(w,\kappa,\beta)}{4k^2\pi^2 + (\log 2)^2(\beta-1)^2}G_r(0) \\ &= \frac{2\rho^2B^2v_1^{2\beta-2}N}{f\eta^2} \times \frac{1}{N^2f^2} \times \sum_{k=1}^{\infty}\frac{C_k(w,\kappa,\beta)}{4k^2\pi^2 + (\log 2)^2(\beta-1)^2}.\end{aligned}$$

Since we have normalized the power spectrum so that $G_c(0) = 1$, the height of the off-zero peak is then the ratio of the absolute heights of the off-zero and zero peaks, which is given by

$$H = \frac{H_{\text{off-zero}}}{H_{\text{zero}}} \approx \frac{C_1(w,\kappa,\beta)N^2}{2\pi^2(4\pi^2 + (\log 2)^2(\beta-1)^2)\sum_{k=1}^{\infty}\frac{C_k(w,\kappa,\beta)}{4k^2\pi^2+(\log 2)^2(\beta-1)^2}}.$$

Since $(\log 2)^2(\beta - 1)^2 \ll 4\pi^2$, the height of the off-zero peak can be simplified as

$$H \approx \frac{C_1(w,\kappa,\beta)N^2}{2\pi^2 C(w,\kappa,\beta)},$$

where

$$C(w,\kappa,\beta) = \sum_{k=1}^{\infty}\frac{C_k(w,\kappa,\beta)}{k^2}.$$

Therefore, the height H is also proportional to N^2. We emphasize that while this conclusion is derived for unstable gene products, it also holds for all gene products, according to our simulations.

D. Technical details for Fig 4

In (a)-(c), the model parameters are chosen as $B = 0.5, \alpha_0 = \alpha_1 = 1, d = \eta f$. The parameters ρ and a are chosen so that $\langle n\rangle = 200$ and $\langle V\rangle = 4$. The remaining parameters are chosen as $N = 50, N_0 = 21, \beta = \kappa = 1, \eta = 0.5$ for (a), $N = 50, N_0 = 21, \beta = 0, \kappa = \sqrt{2}, \eta = 0.5$ for (b), and $N = 80, N_0 = 60, \beta = 1, \kappa = 2, \eta = 5$ for (c).

In (d)-(f), the model parameters are chosen as $N = 50, B = 1, \alpha_0 = \alpha_1 = 1, d = \eta f$. The parameters ρ and a are chosen so that $\langle n\rangle = 100$ and $\langle V\rangle = 1$. The remaining parameters are chosen as $N_0 = 21, \eta = 1$ for (a), $N_0 = 21, \kappa = 2$ for (b) and $\beta = 0, \eta = 1$ for (c). While we have assumed the adder strategy in the simulations, similar results also hold for other control strategies.

In (a)-(f), the growth rate g is determined so that $f = 0.1$.

E. Technical details for Fig 5

In (e), the model parameters are chosen as $N = 50, N_0 = 23, \rho = 17, B = 1, \beta = 1, \kappa = 2, d = 0.1, \eta = 1$. The parameter a is chosen so that $\langle V\rangle = 1$. The strengths α_0 and α_1 of size control are chosen as $\alpha_0 = \alpha_1 = 1$ for the upper panel (adder), $\alpha_0 = 0.5, \alpha_1 = 2$ for the middle panel (timer-sizer), and $\alpha_0 = 2, \alpha_1 = 0.5$ for the lower panel (sizer-timer). The growth rate g is determined so that $f = 0.1$.

F. Technical details for Fig 6

In (a)-(c), the model parameters are chosen as $N = 50, B = 1, \kappa = 2, \alpha_0 = \alpha_1 = 1, d = \eta f$. The parameters ρ and a are chosen so that $\langle n\rangle = 100$ and $\langle V\rangle = 1$. The remaining parameters are chosen as $N_0 = 25, \beta = 0, \eta = 0$ for (a), $N_0 = 10, \beta = 0, \eta = 3$ for (b), and $N_0 = 16, \beta = 1, \eta = 10$ for (c).

In (d), the model parameters are chosen as $N = 30, N_0 = 11, \rho = 66, B = 1, \beta = 1, \kappa = 2, d = 1, \eta = 10$. The parameter a is chosen so that $\langle V\rangle = 1$. The strengths α_0 and α_1 of size control are chosen as $\alpha_0 = \alpha_1 = 1$ for the blue curve, $\alpha_0 = 0.5, \alpha_1 = 2$ for the red curve, and $\alpha_0 = 2, \alpha_1 = 0.5$ for the green curve.

In (e),(f), the model parameters are chosen as $N = 30, N_0 = 18, \alpha_0 = \alpha_1 = 1, d = \eta f$. The parameters ρ and a are chosen so that $\langle n\rangle = 100$ and $\langle V\rangle = 1$. The remaining parameters are chosen as $B = 0.2, \eta = 10$ for (e) and $\beta = 1, \kappa = 2$ for (f).

In (a)-(f), the growth rate g is determined so that $f = 0.1$.

G. Parameter inference using synthetic data

Our model is complex due to the coupling between gene expression dynamics, cell volume dynamics, and cell cycle events. A natural question is whether all the parameters involved in the model can be inferred accurately. In fact, parameter inference for similar models has been made in our previous papers — the parameters related to cell volume dynamics have been inferred in *E. coli* [6] and fission yeast [7] using the method of distribution matching, and the parameters related to gene expression dynamics have be estimated in *E. coli* [8] using the method of power spectrum matching. Suppose that the time course data of cell size and gene expression (mRNA or protein abundance) can be measured along a cell lineage. Next we briefly introduce the parameter inference method for our model and validate it using synthetic data.

1) Estimation of g. Note that the cell volume at birth V_b, the cell volume at division V_d, and the cell cycle duration T in each generation can be easily extracted from the lineage data. Since the growth of cell volume is exponential, $g = \langle \log(V_d/V_b)/T\rangle$ gives an estimate of the growth rate, where the angled brackets denote the average over generations.

2) Estimation of α_0 and α_1. Suppose that the cell volume at replication V_r can also be measured, e.g. fluorescent probes were used in [9] to label individual nuclei in the G_1 phase red and those in the G_2-S-M phase green. In Methods, we prove that when the variability in cell size is small, the volumes at birth, replication, and division are linearly related by $V_r - 2^{w(1-\alpha_0)}V_b = \epsilon_0$ and $V_d - 2^{(1-w)(1-\alpha_1)}V_r = \epsilon_1$, where $w = \langle \log_2(V_r/V_b)\rangle$ is the fraction of cell cycle before replication, ϵ_0 is a noise term independent of V_b, and ϵ_1 is a noise term independent of both V_b and V_r. Then the slope of the regression line of V_r on V_b gives an estimate of $2^{(1-\alpha_0)w}$, from which α_0 can be determined. Similarly, the slope of the regression line of V_d on V_r gives an estimate of $2^{(1-\alpha_1)(1-w)}$, from which α_1 can be determined.

If V_r cannot be obtained directly from lineage measurements, then we can use step 6) below to estimate w from which the volume at replication can be estimated as $V_r = 2^w V_b$.

3) Estimation of N_0 and N. Recall that the generalized added volumes $\Delta_0 = V_r^{\alpha_0} - V_b^{\alpha_0}$ and $\Delta_1 = V_d^{\alpha_1} - V_r^{\alpha_1}$ have Erlang distributions with shape parameters N_0 and $N_1 = N - N_0$, respectively. Once α_0 and α_1 have been estimated, N_0 and N_1 can be inferred as the inverse CV squared of Δ_0 and Δ_1, respectively.

4) Estimation of a. The last parameter related to cell volume dynamics is the proportionality constant a for the transition rate between cell cycle stages. This parameter can be determined by an optimal fit of the experimental to the theoretical/simulated doubling time distribution using the least squares criterion. Specifically, we can infer a by solving the following optimization problem:

$$\min_{\alpha} \sum_{i=1}^{L} |p(x_i) - \hat{p}(x_i)|^2, \tag{21}$$

where $p(x)$ is the theoretical/simulated doubling time distribution, $\hat{p}(x)$ is the sample doubling time distribution obtained from experiments, x_i are some reference points, and L is the number of bins chosen.

5) Estimation of d and β. The degradation rate d can be determined by measuring the half-life of the gene product [10]. For stable proteins, we may simply take $d = 0$ [8, 11]. The degree β of balanced biosynthesis can be determined by *a priori* knowledge. If the mRNA number in a population of cells scales linearly with cell volume, we can take $\beta = 1$. If the mRNA number in G_1 (before replication) or G_2 (after replication) phase does not scale with cell volume, we can take $\beta = 0$ [10].

6) Estimation of ρB and κ. Since the burst frequency increases from ρ to $\kappa\rho$ upon replication, we can fit the gene expression data within each cell cycle by the solution of the following mean-field differential equation:

$$\frac{\mathrm{d}}{\mathrm{d}t}\hat{n}(t) = \begin{cases} \rho B V(t)^{\beta} - d\hat{n}(t), & \text{if } 0 \leq t < wT, \\ \kappa\rho B V(t)^{\beta} - d\hat{n}(t), & \text{if } wT \leq t \leq T, \end{cases} \tag{22}$$

where t is the cell age. By fitting the time course data $n(t)$ to the approximation $\hat{n}(t)$, we obtained the least squares estimates of ρB and κ by minimizing the (squared) distance $\sum_{t=0}^{M-1}[n(t) - \hat{n}(t)]^2$ between the two, where M is the number of time points measured within a cell cycle.

If the proportion w of cell cycle before replication cannot be obtained in step 2), then we can estimate ρB, w, and κ simultaneously by minimizing $\sum_{t=0}^{M-1}[n(t) - \hat{n}(t)]^2$, from which w can be determined.

7) Estimation of ρ and B. Note that ρB has been estimated in 6). Finally, we can estimate ρ and B separately by an optimal fit of the experimental to the theoretical/simulated copy number distribution of the gene product using the least squares criterion.

To verify the effectiveness of our method, we use our model to generating synthetic lineage data of cell volume and gene expression. We then perform parameter inference by fitting the noisy data to our model. The parameters input to the synthetic data and the parameters estimated using the above method are given in Table A, where three sets of input parameters are chosen to cover different biosynthesis patterns (balanced and non-balanced), different degrees of burstiness (small, intermediate, large), different size control strategies (adder, timer-sizer, and sizer-timer), different gene product stability (stable, intermediate, and unstable), and different degrees of dosage compensation (perfect, intermediate, and no). It can be seen that fitting the lineage data to the model leads to accurate estimation of all model parameters. Note that here d and β are determined by *a priori* knowledge or additional experiments and do not need to be estimated.

first set of model parameters						
	g	ρ	B	κ	β	d
input parameters	1	500	0.2	1	1	0
inferred parameters	1.00 ± 0.001	490.56 ± 43.02	0.22 ± 0.03	0.97 ± 0.08	1	0
	N	N_0	α_0	α_1	a	w
input parameters	20	10	2	0.5	840	0.54
inferred parameters	20.08 ± 1.59	10.16 ± 1.19	2.08 ± 0.44	0.54 ± 0.11	826.69 ± 168.07	0.54 ± 0.01
**second set of model parameters**						
	g	ρ	B	κ	β	d
input parameters	1	150	1	1.5	1	1
inferred parameters	1.00 ± 0.001	153.72 ± 12.95	1.07 ± 0.13	1.56 ± 0.13	1	1
	N	N_0	α_0	α_1	a	w
input parameters	40	16	1	1	60	0.48
inferred parameters	39.78 ± 3.41	16.10 ± 1.73	1.00 ± 0.17	1.03 ± 0.12	65.22 ± 12.62	0.48 ± 0.01
**third set of model parameters**						
	g	ρ	B	κ	β	d
input parameters	1	80	5	2	0	5
inferred parameters	1.00 ± 0.001	83.96 ± 7.17	4.92 ± 0.38	1.94 ± 0.21	0	5
	N	N_0	α_0	α_1	a	w
input parameters	60	18	0.5	2	10	0.45
inferred parameters	60.63 ± 4.83	17.65 ± 2.12	0.53 ± 0.09	1.99 ± 0.21	10.54 ± 2.15	0.46 ± 0.01

Table A. **Parameter inference using synthetic data.** The lineage data of cell volume and gene expression are generated from the model. For each set of model parameters, we generate synthetic data simulating 50 cell lineages, each composed of 200 generations. The frequency of sampling is chosen so that on average, 50 points are measured for each lineage, on par with recent mother machine experiments [11]. The value in each cell shows the mean and standard deviation of the estimated parameter computed over 50 cell lineages.

References

[1] Paulsson, J. & Ehrenberg, M. Random signal fluctuations can reduce random fluctuations in regulated components of chemical regulatory networks. *Phys. Rev. Lett.* **84**, 5447 (2000).

[2] Jia, C., Zhang, M. Q. & Hong, Q. Emergent Lévy behavior in single-cell stochastic gene expression. *Phys. Rev. E* **96**, 040402(R) (2017).

[3] Chen, X. & Jia, C. Limit theorems for generalized density-dependent Markov chains and bursty stochastic gene regulatory networks. *J. Math. Biol.* **80**, 959–994 (2020).

[4] Jia, C., Yin, G. G., Zhang, M. Q. *et al.* Single-cell stochastic gene expression kinetics with coupled positive-plus-negative feedback. *Phys. Rev. E* **100**, 052406 (2019).

[5] Friedman, N., Cai, L. & Xie, X. S. Linking stochastic dynamics to population distribution: an analytical framework of gene expression. *Phys. Rev. Lett.* **97**, 168302 (2006).

[6] Jia, C., Singh, A. & Grima, R. Cell size distribution of lineage data: analytic results and parameter inference. *iScience* **24**, 102220 (2021).

[7] Jia, C., Singh, A. & Grima, R. Characterizing non-exponential growth and bimodal cell size distributions in fission yeast: An analytical approach. *PLoS Comput. Biol.* **18**, e1009793 (2022).

[8] Jia, C. & Grima, R. Frequency domain analysis of fluctuations of mRNA and protein copy numbers within a cell lineage: theory and experimental validation. *Phys. Rev. X* **11**, 021032 (2021).

[9] Sakaue-Sawano, A. *et al.* Visualizing spatiotemporal dynamics of multicellular cell-cycle progression. *Cell* **132**, 487–498 (2008).

[10] Skinner, S. O. *et al.* Single-cell analysis of transcription kinetics across the cell cycle. *Elife* **5**, e12175 (2016).

[11] Tanouchi, Y. *et al.* A noisy linear map underlies oscillations in cell size and gene expression in bacteria. *Nature* **523**, 357–360 (2015).
