# Supplementary material for: Concentration fluctuations in growing and dividing cells: Insights into the emergence of concentration homeostasis
Source: PLoS Comput Biol. 2022 Oct 4;18(10):e1010574. doi: 10.1371/journal.pcbi.1010574 (PMC9565450; doi:10.1371/journal.pcbi.1010574)
Supplement: Attachment — Submitted filename: 126102_1_rebuttal_2403562_rdlfqk.pdf [file pcbi.1010574.s010.pdf]

# Response to Referee 1

Thank you for your valuable comments and suggestions which have helped us greatly in improving the quality of this paper. Below we provide a point-by-point response to each comment. All modifications made in the revised manuscript are shown in blue.

1. *This manuscript constructs a gene expression model with various factors. Specifically, the effect of cell size on gene expression is considered, which is often ignored by previous studies.*

*One interesting finding is that the absolute number of the gene products and the concentration can have different distributions. Some predictions of the models are validated by experimental data on E. coli and yeast.*

*This manuscript uses the mean-field approximation for cell volume, which has good accuracy when the number of stages is large. The usage of the power spectrum has a satisfactory effect on studying the concentration oscillation.*

**Response:** Thank you for the positive comments.

2. *Overall the paper was very difficult to follow and digest easily because of all the different factors and mechanisms invoked. It is mainly an issue of providing sufficient details for each of the factors and organizing them in a systematic and logical way. Although there is a supplementary appendix, it was hard to keep track of all the elements in the main manuscript. Perhaps something like Fig 1 of the Appendix can be presented in the main body to outline all the ingredients and how they affect each other.*

**Response:** In the revised manuscript, we moved Supplementary Fig. S1 in the previous version into the main text to outline all the ingredients and how they affect each other (see page 8, Fig. 2). Moreover, we provided many details for each of the biological factors and tried to organize them in a more systematic and logical way (see pages 3-7).

3. *It might be good to provide a more detailed description of the goal (studying gene product number and concentration under different parameters) after introducing the full and the reduced models. A table of symbols would also be helpful.*

**Response:** In the revised manuscript, we added a table explaning the meaning of all model parameters (see page 4, Table 1). Moreover, we provided a detailed description of the goal of the present paper after introducing the full and reduced models (see page 7).

4. *Some technical details in the Methods section are in fact helpful in understanding the conclusions. They can be moved to the Results section.*

**Response:** In the revised manuscript, we moved many technical details in Methods and Supplementary Notes to the main text to help the readers better understand the conclusions (see pages 5-10).

5. *One concern is that the central concept of this manuscript, "stage", is not thoroughly discussed. This concept should have some significant biological meaning, not just be coined for mathematical convenience.*

**Response:** In the revised manuscript, we explained in detail the biological meaning of the effective cell cycle stages (see page 4). Specifically, recent studies have revealed that in many cell types, the accumulation of some activator to a critical threshold is used to promote mitotic entry and trigger cell division, a strategy known as activator accumulation mechanism. In *E. coli*, the activator was shown to be FtsZ; in fission yeast, it is believed to be a protein upstream of Cdk1, the central mitotic regulator, such as Cdr2, Cdc25, and Cdc13. Biophysically, the $N$ effective stages can be understood as different levels of the key activator. Moreover, we pointed out that the power law form for the rate of cell cycle progression may come from cooperativity of the key activator that triggers cell division.

6. *Fig. 1(b) is a little strange. For the left panel, the x-axis (stage) is discrete, then the volume (y-axis) should be a step function, not a straight red line.*

**Response:** In the revised manuscript, we added some red dots in the stage-volume plot to show the dependence of the mean cell volume $v_k$ on cell cycle stage $k$ for the mean-field model (see page 3, Fig. 1). Moreover, we emphasized that the joining of these dots by a straight red line is simply a guide to the eye.

**Significance**

7. *The main advance is a more complete model of gene expression under more realistic organism growth conditions.*

**Response:** Thank you for acknowledging the results of the manuscript.

# Response to Referee 2

Thank you for your valuable comments and suggestions which have helped us greatly in improving the quality of this paper. Below we provide a point-by-point response to each comment. All modifications made in the revised manuscript are shown in blue.

0. *Jia et al. introduce a modeling framework to represent stochastic gene expression, with an explicit representation of cell volume growth, cell cycle progression (and its dependency on cell volume) and gene dosage compensation. The model is very elegant and general in that it can represent a variety of situations, simply as a matter of parametrization. Under a simplifying assumption, the authors derive a number of metrics (include stationary distribution of gene product and power spectrum of gene product fluctuation dynamics), for both absolute number and concentration of gene product molecules. They use their model and derivations to examine under which conditions cell can achieve homeostasis in the concentration of the expressed gene product, despite changes in cell volume and gene copy number following replication. They also present and discuss the conditions giving rise to specific features (i.e. bimodality in stationary distribution, peak in power spectrum) and examine these features in experimental data to conclude to infer the underlying homeostasis strategies.*

*The model is rather general and powerful. The simplifying assumption seems reasonable (and the authors investigate to some extent its limitations, i.e. Fig. 2). The conclusions are overall convincing.*

**Response:** Thank you for the positive comments.

### Major comments

1. *My main concern is that the metrics that the authors use to assess concentration homeostasis (i.e. the $\gamma$ parameter and the presence / absence of peak in power spectrum) do not seem quite appropriate to describe how much variability / fluctuations in concentration are driven by cell cycle effects. Indeed, the $\gamma$ parameter measures how much the *average* concentration in each cell cycle stage varies throughout the cell cycle. However, this variability should be compared to the total variability due to both cell cycle effects and stochastic bursting dynamics. A given level of cell-cycle dependency (say $\gamma = 0.2$) could be very visible if gene expression is weakly noisy (e.g. $B$ low and $\langle n \rangle$ high) and completely invisible is gene expression is highly bursty (large $B$ and small $\langle n \rangle$). In the latter situation, cell-cycle effects would be meaningless for the cell to minimize. In essence, reusing the authors notations, I think $\gamma/\phi^{1/2}$, would be a more relevant metric to observe.*

**Response:** In the revised manuscript, we showed that the total concentration noise $\phi$ can be decomposed as $\phi = \phi_{\text{ext}} + \phi_{\text{int}}$, where $\phi_{\text{ext}}$ is the extrinsic noise which characterizes the fluctuations between different stages due to cell cycle effects and $\phi_{\text{int}}$ is the intrinsic noise which characterizes the fluctuations within each stage due to stochastic bursty synthesis and degradation of the gene product (see page 11). Based on the above decomposition, we introduced a new metric $\gamma = \phi_{\text{ext}}/\phi$, which characterizes the accuracy of concentration homeostasis. Clearly, the new metric $\gamma$ reflects the relation contribution of cell cycle effects in the total concentration variability. All discussions about concentration homeostasis are based on the new metric $\gamma$ in the revised manuscript. Moreover, all figures have been updated by using this new metric.

2. *Similarly, when inspecting the peak in the power spectrum, the weight of the Lorentzian function(s) creating the peak, should be compared to the stationary component ($\lambda_N$, $u_N$ in the authors' notations).*

**Response:** We cannot quite understand why the weights $u_k$ of the Lorentzian functions should be compared to the stationary component $u_N$. In fact, all the weights $u_k$ except $u_N$ are actually complex numbers and we are not so sure about the meaning of $u_k/u_N$. However in the revised manuscript, we emphasized that the power spectrum $G(\xi)$ is normalized so that $G(0) = 1$ throughout the paper (see page 13). To better understand concentration oscillations and its relation to homeostasis, we depicted both $\gamma$ and $H$ as a function of $B$ and $\langle n\rangle$ (see Supplementary Fig. S5). As expected, the off-zero peak becomes lower as $B$ increases and as $\langle n\rangle$ decreases since both of them correspond to an increase in concentration fluctuations which counteracts the regularity of oscillations; noise above a certain threshold can even completely destroy oscillations. Furthermore, we found that $\gamma$ and $H$ have similar dependence on $B$ and $\langle n\rangle$. This again shows that the occurrence of concentration oscillations is intimately related to the visibility of cell cycle effects in concentration fluctuations.

*A complementary analysis including these two points and a discussion the relative contribution of cell-cycle effects and bursting dynamics in the total variability/fluctuation of concentrations would be important to include.*

**Response:** In the revised manuscript, we made some complementary analysis and discussion about the relative contribution of cell cycle effects and stochastic birth-death dynamics in the total variability of concentrations (see pages 11-14).

### Minor comments

3. *The dashed line on Fig. 3a is defined as $\kappa = \sqrt{2}^{1-\beta}$. First is this empirical or does it come from a derivation? Second, it seems incomplete since it should depend on $w$. Intuitively, this line should correspond to the value of $\kappa$ that would best mimic balanced biosynthesis in the case where $\beta \neq 1$. In other words, $\kappa$ should be so that*

$$\langle \rho B'/V(t)\rangle_{\mathrm{prereplication}} = \langle \kappa\rho B'/V(t)\rangle_{\mathrm{postreplication}},$$

*which yields*

$$\kappa = 2^{w(1-\beta)} * (w-1)/w * [2^{w(\beta-1)} - 1]/[2^{(1-w)(\beta-1)} - 1].$$

*This indeed simplifies into $\kappa = \sqrt{2}^{1-\beta}$ when $w = 0.5$.*

**Response:** Thank you for providing such a beautiful derivation. In the revised manuscript, we added this derivation into the main text (see pages 12-13). Moreover, we also made it clear that this relation can also be obtained from the perspective of power spectrum (see page 14).

4. *$\eta$ is used in the caption of Fig. 2, which is cited on page 4. But it is defined only 2 sections later, on page 6.*

**Response:** In the revised manuscript, we gave the definition of $\eta$ in both Table 1 and the caption of Fig. 3 (Fig. 2 in the old version). Please see page 4, Table 1 and page 9, Fig. 3.

5. *$w$ is used in the main text, but only defined in the caption of Fig. 3.*

**Response:** In the revised manuscript, we gave the definition of $w$ in both Table 1 (see page 4) and on page 7.

6. *$w$ is defined as "the proportion of cell cycle before replication". Is this in terms of cell cycle stages (i.e. $w = N_0/N$) or actual time?*

**Response:** In the revised manuscript, we made it clear that $w$ represents the proportion of cell cycle duration before replication, which should be distinguished from the proportion $N_0/N$ of cell cycle stages before replication (see page 7). This is because the transition rate between cell cycle stages is an increasing function of cell size, which means that earlier (later) stages have longer (shorter) durations.

7. *Fig. 3 indicates that power spectra are normalized so that $G(0) = 1$, but $G(0) = 10$ on the first two graphs.*

**Response:** Corrected as suggested (see page 12, Fig. 4). Thank you.

8. *Page 11: "bimodality in the concentration distribution is significantly less apparent". I would suggest rephrasing "bimodality in the concentration distribution is absent" since there should be no reference to "significance" and bimodality is either present or absent (binary), not less apparent.*

**Response:** Corrected as suggested. Thank you.

**Referees cross-commenting**

9. *Regarding the comment from reviewer 3 that "a direct validity test should use data sets of at least two types (total, nascent RNA, etc)". I almost made a related comment in my review, but then I held it off: This issue with using nascent RNA data is that their model does not allow an ON state. They assume that gene products are produced in instantaneous bursts, which is a fair assumption if the lifetime of gene products is large compared to the time the gene stays ON. This is ok if the considered "gene products" are mRNA or proteins, but not nascent RNAs (for which the lifetime is the time to transcribe the gene). I did not make this comment in the end because I think the model is useful regardless. To comply with reviewer 3's request, maybe the authors could use distributions of mRNA and protein products, but I'm not sure that such data exists (since they need cell-cycle-resolved data).*

**Response:** It is not possible to validate our model with nascent mRNA data because the model in its present form cannot predict nascent mRNA fluctuations. This is because unlike mature mRNA, nascent mRNA cannot be assumed to decay via first-order kinetics. A detailed response is provided below to the original comment made by Referee 3. Regarding the comment on the use of cell-cycle-resolved data measuring mRNA and protein expression – while we agree it would make an excellent test of our model, we could not find such a dataset in the literature. We point out that our model, in its present form, is interesting as it is, as a detailed biological model of mature mRNA and protein number / concentration fluctuations in growing cells. Its predictions are yet to be fully confirmed and hence may stimulate the development of further experimental single-cell studies.

**Significance**

10. *The advance of this paper is essentially technical. The authors present a model that incorporates and unifies previously studied effects (cell volume homeostasis, concentration homeostasis, bursting transcription). There is no major conceptual novelty, but the combination of these different aspects and the derivations that authors present are very valuable and might be applicable to interpret data in various species.*

**Response:** Thank you for acknowledging the results of the manuscript.

# Response to Referee 3

Thank you for your valuable comments and suggestions which have helped us greatly in improving the quality of this paper. Below we provide a point-by-point response to each comment. All modifications made in the revised manuscript are shown in blue.

0. *The manuscript analyses a phenomenological model of stochastic gene expression. The model couples bursty transcription with cell growth, division and DNA replication. The cell cycle is divided into a large number of stages whose exponential lifetimes depend on the cell volume. It is argued that concentrations of gene products are distributed according to mixed Gamma distributions, whereas the copy numbers follow mixed negative binomial distributions. The number of modes can be different for concentrations and copy numbers, for instance the copy numbers can be unimodal while concentrations are bimodal. The case when the mean concentration does not depend on the cell cycle stage is called perfect homeostasis. It is argued that perfect homeostasis leads to Gamma distribution of the gene product concentration and that deviations from a Gamma distributions result mainly from deviations of the concentration from perfect homeostasis. It is also proposed that concentration homeostasis is difficult to obtain. These qualitative predictions of the model are tested using two data sets, one for E.coli and another for fission yeast.*

**Response:** Thank you for acknowledging the results of the manuscript.

### Major comments

1. *A huge number of states called "cell cycle stages" have exponential life times. On my opinion, this sequence of stages is just a technicality for keeping the model within a discrete Markovian framework. More natural choices are possible, such as piecewise deterministic Markov processes, age structured diffusions, etc. The biological significance (if there is any) of such states should be explained.*

**Response:** In the revised manuscript, we explained in detail the biological meaning of the effective cell cycle stages (see page 4). Specifically, recent studies have revealed that in many cell types, the accumulation of some activator to a critical threshold is used to promote mitotic entry and trigger cell division, a strategy known as activator accumulation mechanism. In *E. coli*, the activator was shown to be FtsZ; in fission yeast, it is believed to be a protein upstream of Cdk1, the central mitotic regulator, such as Cdr2, Cdc25, and Cdc13. Biophysically, the $N$ effective stages can be understood as different levels of the key activator. Moreover, we pointed out that the power law form for the rate of cell cycle progression may come from cooperativity of the key activator that triggers cell division.

2. *The timescales of stochastic gene expression are not correctly taken into account. It is considered that during an exponential stage the bursting approximation describes gene expression in terms of Gamma distributions for concentrations and in terms of negative binomial distributions for copy numbers. This approximation is only valid if the lifetime of a stage is much larger than the time needed to generate a burst. For RNA, this condition cannot be fulfilled for a large number of states $N$ and/or for two states promoters with a relatively long ON state. For the protein and/or in the case of translational bursting, the condition is even more difficult to fulfil.*

*I agree with the Reviewer 2 that once the master equation accepted the results make sense. But my criticism is different and concerns the master equation itself. In this equation the burst is considered instantaneous, whereas it needs finite time in reality.*

*Concerning nascent mRNA, ON/OFF etc. I disagree. The notion of instantaneous burst with well defined burst size and burst frequency on a stage has a meaning if the lifetime of this stage (which is not mRNA or protein lifetime) is short. The model validity should be clearly stated.*

**Response:** Thank you for pointing out this important issue. When we talk about the validity of the model, we should stick to the full model, instead of the mean-field model. This is because once the full model makes sense, the mean-field model must work well when $N \gg 15$, as we have shown in Fig. 3 and Supplementary Fig. S3. **Hence our reply is based on the validity of the full model.** We will reply to the above comments from the following three aspects.

First, we agree with the referee that in our model, we assume that the gene product is produced in instantaneous bursts with the reaction scheme

$$G \xrightarrow{\rho p^k(1-p)} G + kM, \quad k \geq 1, \quad M \xrightarrow{d} \varnothing, \tag{1}$$

where the mean burst size scales as $V(t)^\beta$. Of course, in reality there is a finite time for the bursts to occur. A more general assumption is that within each cell cycle, the gene expression dynamics is characterized by the following three-stage model:

$$\begin{gathered} G \xrightarrow{\rho} G^*, \quad G^* \xrightarrow{r} G, \quad G^* \xrightarrow{sV(t)^\beta} G^* + M, \quad M \xrightarrow{u} M + P, \\ M \xrightarrow{v} \varnothing, \quad P \xrightarrow{d} \varnothing, \end{gathered} \tag{2}$$

where the first two reactions describe the switching of the gene between an inactive state $G$ and an active state $G^*$, the middle two reactions describe transcription and translation, and the last two reactions describe the degradation of the mRNA $M$ and the protein $P$. Here the synthesis rate of mRNA depends on cell volume via a power law form with power $\beta \in [0, 1]$. Dosage compensation can be modeled by a decrease in the gene activation rate (for each gene copy) from $\rho$ to $\kappa\rho/2$ upon replication. Previous studies have revealed that the bursting of mRNA and protein has different biophysical origins: transcriptional bursting is due to a gene that is mostly inactive, but transcribes a large number of mRNA when it is active ($r \gg \rho$ and $s/r$ is finite), whereas translational bursting is due to rapid synthesis of protein from a single short-lived mRNA molecule ($v \gg d$ and $u/v$ is finite). Under the above timescale separation assumptions, both mRNA and protein are produced in a bursty manner with the reaction scheme described by Eq. (1). The burst frequency for mRNA and protein are both $\rho$ before replication and $\kappa\rho$ after replication. The mean burst size for mRNA is $(s/r)V(t)^\beta$ and the mean burst size for protein is $(su/rv)V(t)^\beta$, both of which have a power law dependence on cell volume (see pages 5-6).

In Supplementary Figs. S1 and S2, we compare the mRNA and protein distributions for the bursty model with the reaction scheme given by Eq. (1) and the three-stage model with the reaction scheme given by Eq. (2), where both models under consideration have a cell cycle and cell volume description. It can be seen that the distributions for the two models are very close to each other under the above timescale separation assumptions with the bursty model being more accurate as $r/\rho$ and $v/d$ increase. Moreover, we find that the accuracy of the bursty model is

insensitive to the value of the number of stages $N$. Here the values of $N$ are chosen so that the ratio of the average time spent in each stage ($T/N$, where $T \approx (\log 2)/g$ is the mean cell cycle duration) and the mean burst duration time ($1/\rho$) ranges from $\sim 0.5 - 2$. This shows that the effectiveness of the bursty model does not require that the lifetime of a cell cycle stage is sufficient long. Due to mathematical complexity, we only focus on the bursty model in the present paper. The consistency between the gene product distributions for the two models justifies our bursty assumption.

Second, while we assume bursty expression here, our model naturally covers non-bursty expression since the latter can be regarded as a limit of the former. Hence all the conclusions in the present paper are applicable to both bursty and non-bursty expression. In the revised manuscript, we emphasized this point (see page 4 for a detailed explanation).

Last but not least, if the lifetime of the gene product is much shorter compared to the lifetime of each cell cycle stage, then the gene expression dynamics will rapidly relax to a quasi-steady state for each stage. In this case, the gene product fluctuations at each stage can be characterized by a gamma distribution in terms of concentrations and by a negative binomial distribution in terms of copy numbers, and hence the distribution of concentrations (copy numbers) for a population of cells is naturally a mixture of $N$ gamma (negative binomial) distributions. However, the powerfulness of our analytical distribution (see page 10, Eq. (8)) is that it serves an accurate approximation when $N \gg 1$ without making any timescale assumptions. The effectiveness of our analytical distributions is validated in Supplementary Fig. S3 for three different cases: (i) the degradation rate $d$ of the gene product is much smaller than the cell cycle frequency $f$; (ii) $d$ and $f$ are comparable; (iii) $d$ is much larger than $f$. In the revised manuscript, we also emphasized these points (see page 10).

3. *DNA replication is a stochastic event and does not occur after a fixed number of exponential stages as it is considered in this model.*

*Concerning replication: in the model this occurs after exactly $N_0$ steps. In reality, replication occurs somewhere between the start of S and $G_2$/M. $N_0$ is in fact a random variable. Probably a new mean field assumption is needed here with some justification, but I have seen nothing in the paper.*

**Response:** We agree with the referee that **replication of the whole genome** occurs in the S phase, which occupies a considerable portion of the cell cycle and thus cannot be assumed to occur after a fixed number of exponential stages. However, **our model is for a single gene** and since the replication time of a particular gene is much shorter than the total duration of the S phase, it is reasonable to consider it to be instantaneous. In addition, recent experiments have shown that the time elapsed from birth to replication for a particular gene occupies an approximately proportion of the cell cycle, which is called the stretched cell cycle model. This is also consistent with our assumption that replication of the gene of interest occurs after exactly $N_0$ stages. While replication occurs after a fixed number of stages, nevertheless the time of replication is stochastic since each stage has a random lifetime. In the revised manuscript, we emphasized these points (see pages 4-5).

4. *The results in the Methods were derived heuristically and their relation to the master equation (12) is not explicit (except for the part concerning moments and their power spectrum). Furthermore, one would like to have some*

*estimates of the biases introduced by the mean field approximation.*

*Concerning biases introduced by the mean field approximation: Figure 2 is a numerical simulation, some analytical estimates could be better. As Figure 2 looks rather convincing, I reclassify this as minor comment.*

**Response:** We agree with the referee that the derivation of moments is rigorous, but the derivation of the analytical distribution given in Methods is not rigorous and cannot be directly obtained from the master equation. In the revised manuscript, we emphasized that the analytical distribution is not exact but it serves as a very good approximation (see pages 10 and 22). We showed that the analytical distribution agrees well with stochastic simulations when the number of cell cycle stages $N \geq 15$ (see page 9, Fig. 3 and Supplementary Fig. S3). The logic behind our approximate distribution is that while the gene product may produce complex distribution of concentrations (copy numbers), when the number of cell cycle stages is large, the distribution must be relatively simple within each stage and thus can be well approximated by a simple gamma (negative binomial) distribution (see page 22).

Due to the complexity of our model, it is very difficult to provide any analytical estimates on the bias introduced by the mean-field approximation. Often the bias of an approximation can be estimated when the approximation emerges from a systematic method such as van Kampen's system-size expansion (see Ref. [21]). However, our mean-field model cannot be seen as the zero order term of some expansion and hence it is not possible to calculate the next-order correction which would be needed to estimate the error. However, we have tested very large swathes of parameter space and found that the mean-field approximation always works well when $N \geq 15$ which is the physiologically relevant regime for most types of cells (see discussion on P. 7).

5. *The model is not minimal and depends on a huge number of parameters. It is not clear how these parameters were found and if overfitting was avoided. One may have doubts about the identifiability of the parameter $N$. What difference is between $N = 59$ and $N = 60$ (the value of N for the cyanobacterium)?*

**Response:** In the revised manuscript, we used synthetic data to show that all the model parameters involved in our model (except $d$ and $\beta$ which can be determined based on *a priori* knowledge) can be accurately estimated from cell-cycle resolved lineage data of cell volume and gene expression (see Supplementary Note 7). We provided details of the parameter inference method, compared the input parameters with the estimated ones and verify that they are identifiable (see Supplementary Table 1). We did not use real data to test our inference method because we could not find cell-cycle resolved lineage data for mRNA or proteins. As we noted, this is in principle possible via cell-cycle fluorescent markers. We also note that parameter inference for less detailed but similar models have been made in our previous papers — the parameters related to cell volume dynamics have been inferred in *E. coli* (see Ref. [51]) and fission yeast (see Ref. [52]) using the method of distribution matching, and the parameters related to gene expression dynamics have be estimated in *E. coli* (see Ref. [40]) using the method of power spectrum matching.

Moreover, for our purpose, i.e. to investigate the effect of cell cycle and cell volume on gene expression, we do believe that **our model is minimal**. We captured cell growth with only one parameter $g$, the degree of balanced biosynthesis with one parameter $\beta$ ($\beta = 0$ corresponds to the case where the synthesis rate is independent of cell volume and $\beta = 1$ corresponds to the case where the synthesis rate scales linearly with cell volume), the variability in cell cycle duration with only one parameter $N$, gene replication with only one parameter $N_0$, gene

dosage compensation with only one parameter $\kappa$ ($\kappa = 1$ corresponds to perfect dosage compensation and $\kappa = 2$ corresponds to no dosage compensation), and the variation of size control strategy across the cell cycle with two parameters $\alpha_0$ and $\alpha_1$ ($\alpha_i \to 0$ corresponds to timer, $\alpha_i = 1$ corresponds to adder, and $\alpha_i \to \infty$ corresponds to sizer). The biological meaning of the cell cycle stages were clarified in the revised manuscript (see page 4). For our purpose, we believe that our model cannot be simpler.

6. *The authors should make clear which cell biology aspects are important, which are less important, and which were neglected in the context of their problem. Thus, in their model, cell cycle acts on gene expression mainly by duplication of burst sources and thus by increase of burst frequency after replication. Another important source of gene expression variability during the cycle, the mitotic transcription repression, is neglected.*

**Response:** In the revised manuscript, we clarified which cell biology aspects are important for gene expression dynamics (see page 17). Specifically, in our model, cell cycle and cell volume act on gene expression mainly by (i) the dependence of the burst size on cell volume; (ii) the increase in the burst frequency upon replication; (iii) the change in size control strategy upon replication; (iv) the partitioning of molecules at division. Point (iv) strongly affects copy number fluctuations, while it has little influence on concentration fluctuations. In addition, in the revised manuscript, we also elucidated the limitations of our model including mitotic transcription repression and others (see pages 19-20).

7. *The validity test of the model is indirect. It was tested that the concentration distribution deviates from Gamma and that the deviation correlates positively to the lack of accuracy of the concentration homeostasis. However, many models can have this behaviour. A direct validity test should use data sets of at least two types (total, nascent RNA, etc.) allowing direct estimates of some model parameters (such as burst size and frequency using nascent RNA).*

*Concerning parsimony, I think that the authors should test it. Are all the parameters identifiable? Is there any overfitting? They could use parameter uncertainty, comparison of training /testing errors, etc. Some details about the parameter fitting method should be provided.*

**Response:** Regarding the parameter fitting and identifiability we have provided a detailed response to a previous comment above.

However we emphasize that for the generation of Fig. 7, we did not need to estimate all model parameters from data. Hence in the previous version of the manuscript, **no such estimation was done** — we simply extracted the homeostasis accuracy $\gamma$, the height $H$ of the off-zero peak of the power spectrum, and the Hellinger distance $D$ of the concentration distribution from its gamma approximation directly from data.

Finally, we point out that our model can be used to predict the dynamics of mature mRNAs, but it cannot be used to describe the dynamics of nascent mRNAs. This is because nascent mRNAs do not decay via a first-order reaction but their removal, i.e. their detachment from the gene which leads to mature mRNA, is better approximated by a reaction with a fixed decay time. This models the elongation time of nascent transcripts which does not suffer from much noise because the RNAP velocity is to a good approximation constant along the gene. See e.g. the following

two papers for details:
H. Xu, S. O. Skinner, A. M. Sokac, I. Golding, Stochastic kinetics of nascent RNA. *Phys. Rev. Lett.* 117, 128101 (2016).
S. Braichenko, J. Holehouse, R. Grima. Distinguishing between models of mammalian gene expression: telegraph-like models versus mechanistic models. *J. R. Soc. Interface* 18, 20210510 (2021).

Because of the fixed delay, the delay telegraph model (the telegraph model with a delayed degradation reaction) is non-Markovian and very different from the usual Markovian telegraph model which describes the dynamics of mature mRNA within each cell cycle. See e.g. the Supplementary Information of the following paper:
X. Fu, et al. Accurate inference of stochastic gene expression from nascent transcript heterogeneity. bioRxiv (2021).

Given the mathematical complexity introduced by a fixed delay, using it to describe the dynamics of nascent mRNA within each cell cycle leads to a non-Markovian model that is even more analytically intractable than the present one for mature mRNA. While an interesting research question, this is clearly far removed from the scope of our current manuscript.

**Minor comments**

8. *The introduction could be more pedagogical. Right now it is just an accumulation of loosely related and sometimes abruptly introduced statements. For instance, we understand that the authors want to oppose their approach to other extant approaches. However, extant approaches should be better reviewed, some of them are aged structured and perfectly suited for analysing cell cycle data. It would be useful for the reader that an example of observation explained by their model and not explained by other models (age structured or not) is discussed in detail. The model of this work does not explain size control, it just assumes that this holds, and does not discuss cell population aspects. A more nuanced positioning of this approach with respect to the literature would be useful for judging its value.*

**Response:** In the revised manuscript, we rewrote the introduction part to make it more pedagogical (see pages 1-2). In particular, we compared three popular models describing the cell size dynamics and the associated size homeostasis. The advantages and disadvantages of the three models were discussed.

9. *The meaning of $N$ should be discussed from the very start when the model is introduced.*

**Response:** In the revised manuscript, we explained in detail the biological meaning of the effective cell cycle stages (see page 4). Specifically, recent studies have revealed that in many cell types, the accumulation of some activator to a critical threshold is used to promote mitotic entry and trigger cell division, a strategy known as activator accumulation mechanism. In *E. coli*, the activator was shown to be FtsZ; in fission yeast, it was believed to be a protein upstream of Cdk1, the central mitotic regulator, such as Cdr2, Cdc25, and Cdc13. Biophysically, the $N$ effective stages can be understood as different levels of the key activator. Moreover, we pointed out that the power law form for the rate of cell cycle progression may come from cooperativity of the key activator that triggers cell division.

10. *The authors call constitutive expression the situation when the mean copy number does not depend on the volume. This choice should be clarified as in general constitutive as opposed to specific, localised or transitory expression refers to non-regulated gene expression. It seems to me that in this context, expression is only partially constitutive (independent on the volume).*

**Response:** In the present paper, constitutive expression means that the gene product is produced one at a time and is not produced in a bursty manner. It does not mean that the mean copy number does not depend on the volume. In the revised manuscript, we provided a more detailed discussion about how constitutive expression can be viewed as a limit of bursty expression (see page 4).

11. *In figure 1b and for exponential growth the y axis should be log(volume) instead of volume. The mean field approximation is called both "of novel type" (Discussion) and "which has a long history of successful use in statistical physics" (p4). If something is novel, then one should clearly explain why.*

**Response:** In fact, the y-axis in Fig. 1(b) should be volume instead of log(volume). This is because the x-axis represents the cell cycle stage instead of the real time. Note that for the adder strategy ($\alpha_0 = \alpha_1 = 1$), it follows from Eq. (3) on page 7 that the mean cell volume at stage $k$ is $v_k = v_1 + (k-1)M_0/N_0$, which linearly depends on $k$. This explains why the red curves in Fig. 1(b) are straight lines instead of exponential curves.

In the revised manuscript, we also explained why the mean-field approximation used is novel (see page 7). Specifically, we pointed out that the mean-field approximation is not made for the whole cell cycle, rather we make the approximation for each stage and thus different stages have different mean cell volumes. This type of piecewise mean-field approximation, as far as we know, is novel and has not been used in the study of concentrating fluctuations before.

12. *The word "cyclo-stationarity" is used with not much definition. If this means just stationary distribution of the gene products why not use just "stationarity" instead. What means "cyclo"? A number of properties were called "rare" but it is not clear on what grounds.*

**Response:** In the revised manuscript, we removed the term "cyclo-stationarity" and simply assumed that the copy number and concentration distributions of the gene product at each cell cycle stage have reached the steady state (see page 8). In addition, for each property that was called "rare", we explained the reasons in detail (see pages 14 and 17).

13. *I did not find a proof that the copy number distribution has less modes than the concentration distribution.*

**Response:** In fact, it is very difficult to prove that the concentration distribution has less modes than the copy number distribution. However, we have tested very large swathes of parameter space and found that the number of modes of the concentration distribution is always less than or equal to that of the copy number distribution. In the revised manuscript, we emphasized this point (see page 16).

## Significance

14. *The strength of this work is that it incorporates in a stochastic gene expression model a number of ideas on size control and dosage compensation that were discussed elsewhere from a cell population point of view. However, the proposed model is based on a number artificial choices that are difficult to justify biologically: a huge number of cell cycle discrete states and inappropriate handling of the timescales characterizing stochastic gene expression. Furthermore, the model is not minimal but depends instead on a huge number of parameters.*

*I found the paper difficult to read and in the results presentation is not suitable for biologists that would need more details on the justification of the modelling choices and on the experimental validation of the model.*

**Response:** All these points have been addressed in previous replies.

15. *For mathematicians, the calculations are rather standard and may seem trivial.*

**Response:** Our model is complex due to the coupling between gene expression dynamics, cell volume dynamics, and cell cycle events. It is far more complex than standard models of gene expression (see e.g. Refs. [2,84,85]) because of the large amount of biology encapsulated in it and we presented a first analytical- and simulation-based analysis of concentration fluctuations when concentration homeostasis is broken.

The computations of many quantities in the present paper are **non-trivial**. First, we showed that the generalized added volumes before and after replication both have an Erlang distribution. Using this property, we computed the mean cell volume in each cell cycle stage which is needed in the mean-field approximation. Furthermore, the computations of the power spectrum of concentration fluctuations are also highly non-trivial. The analytical expression of the power spectrum allows us to precisely determine the onset of concentration homeostasis. While the computations of moments of concentration fluctuations are standard, we used to the moments to construct an analytical concentration distribution which serves as an accurate approximation when $N$ is large. Our concentration distribution is generally valid when concentration homeostasis is broken and goes far beyond recent models for growing cells which require concentration homeostasis and which do not take into account DNA replication, dosage compensation and size control mechanisms that vary with the cell cycle phase (e.g. Ref. [26] ).
