# Supplementary material for: Concentration fluctuations in growing and dividing cells: Insights into the emergence of concentration homeostasis
Source: PLoS Comput Biol. 2022 Oct 4;18(10):e1010574. doi: 10.1371/journal.pcbi.1010574 (PMC9565450; doi:10.1371/journal.pcbi.1010574)
Supplement: Attachment — Submitted filename: response.pdf [file pcbi.1010574.s011.pdf]

Response to Referee 1

Thank you for your valuable comments and suggestions which have helped us greatly in improving the quality of this paper. Below we provide a point-by-point response to each comment. All modifications made in the revised manuscript are shown in blue.

1. *In this revised version of the manuscript by Jia et al., the authors have appropriately addressed all of my comments, except one, for which clarifications or modifications would further improve the manuscript.*

This is regarding the earlier comment I made on the power spectrum analysis in my first review. This comment was motivated by the same idea as the one just before, i.e. that the metric for quantifying homeostasis should be a dimensionless measure of the relative contribution of oscillations to the total fluctuations (which includes both a cell-cycle-driven component and a stochastic-bursting-driven component). If the metric you use is the height of the peak, it should be normalized to something that somehow measures the total fluctuations. I understand now that $G(0)$ *is normalized to 1 even in the calculations (I thought it was just on the plots) and realize that* $C(\beta, \kappa, \omega)$ *is already dimensionless. So you might already be doing what I have in mind. But in that case, you should motivate this* $G(0) = 1$ *normalization and explain more intuitively what* $C(\beta, \kappa, \omega)$ *really quantifies. You did it very well with* γ *(explaining that* ϕ *has two components and that* γ *quantifies the relative contribution of the extrinsic oscillations* ϕ_{ext} *to the total* ϕ*). Something similar for the power spectrum would be a big plus.*

Response: In the revised manuscript, we explained that the power at zero frequency, $G(0)$, characterizes the strength of stochasticity that is not owing to oscillations. Hence, we have normalized the the power spectrum so that $G(0) = 1$ throughout the paper. In this way, the height of the non-zero peak (relative to the power at zero frequency) acts as a measure of the regularity of concentration oscillations (see pages 13-14).

Response to Referee 2

Thank you for your valuable comments and suggestions which have helped us greatly in improving the quality of this paper. Below we provide a point-by-point response to each comment. All modifications made in the revised manuscript are shown in blue.

1. *However, I would encourage the authors to perform an additional round of writing where they clearly separate assumptions from their justifications, and from validation attempts with published results or experimental data. In particular, this refers to the points 6.7 and 14 raised by reviewer 3. I also note that the processes considered in this study are not the sole ones that may play a role in concentration homeostasis, and this may emerge e.g. in the discussion.*

Response: In the revised manuscript, we emphasized that there are additional mechanisms that also contribute to concentration homeostasis besides the ones discussed in this paper. For example, in some cell types, both the synthesis and degradation rates of mRNA may be volume-dependent and this is needed to maintain concentration homeostasis because the synthesis rate does not always scale linearly with cell size. In addition, recent studies showed that nuclear polymerase II levels are limiting for transcription and may play central role in maintaining concentration homeostasis, and as well there is likely negative feedback from nuclear mRNA on transcription that couples the kinetics of the various stages of mRNA processing and transport to ensure mRNA concentration homeostasis (see page 19 and references therein).

2. *As specific example, I find that some of the statements made by the authors to justify the model (also in reply to the reviewers) are fragile. For example, it is not true that in E. coli it is established that a threshold accumulation process related to FtsZ sets cell division, and almost surely things are more complex than that. Equally, for yeast, things are complex and not well understood, and surely inhibitor dilution processes were argued to be very important at least for some cell cycle stages.*

Response: In the revised manuscript, we deleted the incorrect statement that a threshold accumulation process related to FtsZ sets cell division in *E. coli*. Moreover, we have also emphasized that inhibitor dilution mechanisms, such as the dilution of Whi5 in budding yeast, may also be used for size control (see page 4).

3. *Additionally, for the question of the model of (single-gene) replication timing I agree with the referee that the question is complex (the authors may want to check PMID for a model of stochastic replication timing in yeast, and PMID: 34795646 - and references therein - for E coli).*

Response: In the revised manuscript, we added some references about the modelling of stochastic replication timing. Moreover, we also emphasized that the timing of replication in bacteria is much more complicated since there may be multiple and overlapping rounds of replication per division cycle (see page 5 and references therein).
